# Dominant *ACO2* mutations are a frequent cause of isolated optic atrophy

**DOI:** 10.1093/braincomms/fcab063

**Published:** 2021-04-07

**Authors:** Majida Charif, Naïg Gueguen, Marc Ferré, Zouhair Elkarhat, Salim Khiati, Morgane LeMao, Arnaud Chevrollier, Valerie Desquiret-Dumas, David Goudenège, Céline Bris, Selma Kane, Jennifer Alban, Stéphanie Chupin, Céline Wetterwald, Leonardo Caporali, Francesca Tagliavini, Chiara LaMorgia, Michele Carbonelli, Neringa Jurkute, Abdelhamid Barakat, Philippe Gohier, Christophe Verny, Magalie Barth, Vincent Procaccio, Dominique Bonneau, Xavier Zanlonghi, Isabelle Meunier, Nicole Weisschuh, Simone Schimpf-Linzenbold, Felix Tonagel, Ulrich Kellner, Patrick Yu-Wai-Man, Valerio Carelli, Bernd Wissinger, Patrizia Amati-Bonneau, Pascal Reynier, European ION Group, Guy Lenaers

**Affiliations:** 1 Université Angers, MitoLab Team, UMR CNRS 6015 - INSERM U1083, Institut MitoVasc, SFR ICAT, Angers, France; 2 Genetics and Immuno-Cell Therapy Team, Mohammed First University, Oujda, Morocco; 3 Département de Biochimie et Génétique, CHU d'Angers, Angers, France; 4 Laboratory of Genomics and Human Genetics, Institut Pasteur du Maroc, Casablanca, Morocco; 5 Unit of Neurology, Department of Biomedical and NeuroMotor Sciences (DIBINEM), University of Bologna, Bologna, Italy; 6 IRCCS Institute of Neurological Sciences of Bologna, Bellaria Hospital, Bologna, Italy; 7 Moorfields Eye Hospital, London, UK; 8 UCL Institute of Ophthalmology, University College London, London, UK; 9 Cambridge Eye Unit, Addenbrooke’s Hospital, Cambridge University Hospitals, Cambridge, UK; 10 Cambridge Centre for Brain Repair and MRC Mitochondrial Biology Unit, Department of Clinical Neurosciences, University of Cambridge, Cambridge, UK; 11 Centre de référence des Maladies Neurogénétiques, Département de Neurologie, CHU d’Angers, Angers, France; 12 Department of Pediatrics, Competence Center of Inherited Metabolic Disorders, Angers Hospital, Angers, France; 13 Eye Clinic, Sourdille Jules Verne, Nantes, France; 14 National Center for Rare Diseases, Genetics of Sensory Diseases, University Hospital, Montpellier, France; 15 Institute for Ophthalmic Research, Centre for Ophthalmology, University of Tübingen, Tübingen, Germany; 16 Praxis für Humangenetik, Tübingen, Germany; 17 Centre for Ophthalmology, University of Tübingen, Tübingen, Germany; 18 Rare Retinal Disease Center, AugenZentrum Siegburg, MVZ ADTC Siegburg GmbH, Siegburg, Germany; 19 RetinaScience, 53113 Bonn, Germany

**Keywords:** *ACO2*, aconitase 2, mitochondria, Krebs cycle, optic neuropathy

## Abstract

Biallelic mutations in *ACO2*, encoding the mitochondrial aconitase 2, have been identified in individuals with neurodegenerative syndromes, including infantile cerebellar retinal degeneration and recessive optic neuropathies (locus OPA9). By screening European cohorts of individuals with genetically unsolved inherited optic neuropathies, we identified 61 cases harbouring variants in *ACO2*, among whom 50 carried dominant mutations, emphasizing for the first time the important contribution of *ACO2* monoallelic pathogenic variants to dominant optic atrophy. Analysis of the ophthalmological and clinical data revealed that recessive cases are affected more severely than dominant cases, while not significantly earlier. In addition, 27% of the recessive cases and 11% of the dominant cases manifested with extraocular features in addition to optic atrophy. *In silico* analyses of *ACO2* variants predicted their deleterious impacts on ACO2 biophysical properties. Skin derived fibroblasts from patients harbouring dominant and recessive *ACO2* mutations revealed a reduction of ACO2 abundance and enzymatic activity, and the impairment of the mitochondrial respiration using citrate and pyruvate as substrates, while the addition of other Krebs cycle intermediates restored a normal respiration, suggesting a possible short-cut adaptation of the tricarboxylic citric acid cycle. Analysis of the mitochondrial genome abundance disclosed a significant reduction of the mitochondrial DNA amount in all *ACO2* fibroblasts. Overall, our data position *ACO2* as the third most frequently mutated gene in autosomal inherited optic neuropathies, after *OPA1* and *WFS1*, and emphasize the crucial involvement of the first steps of the Krebs cycle in the maintenance and survival of retinal ganglion cells.

## Teaser

By screening European cohorts of individuals with genetically unsolved inherited optic neuropathies, Charif et al. report 61 new cases harbouring variants in *ACO2*, among whom 50 with dominant mutations, emphasizing for the first time the important contribution of *ACO2* monoallelic pathogenic variants to dominant optic atrophy.

## Graphical Abstract

**Graphical Abstract:**
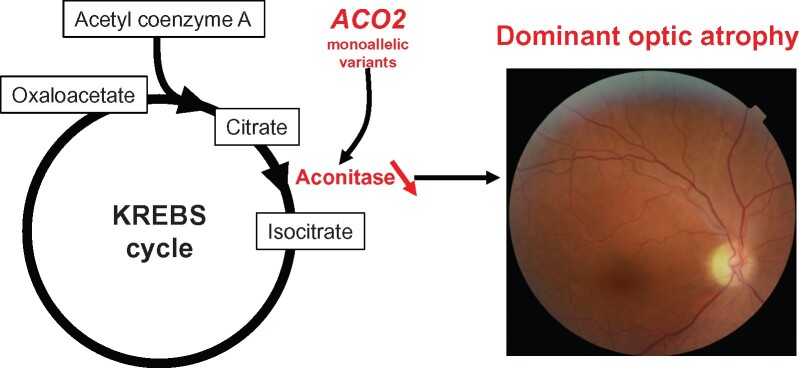

## Introduction

Mitochondrial diseases represent a group of complex pathologies affecting several organs, sometimes presenting with a single symptom, but often combining multiple symptoms.^1^ This complexity depends on the dual genetic contribution of the nuclear and the mitochondrial genome (mtDNA). To date, mutations in all mitochondrial genes and in more than 400 nuclear genes have been reported, sometimes in combinations. In most cases, the mitochondrial respiratory chain driving ATP production is affected, but alterations of alternative mitochondrial mechanisms as metabolic pathways, protein import and membrane dynamics have been identified in many diseases, mainly disrupting neuronal physiology.

Aconitase 2 nuclear gene (*ACO2*; MIM#100850) encoding the mitochondrial aconitase 2 (ACO2; EC# 4.2.1.3) is involved in the reversible isomerization of citrate into iso-citrate, through the *cis-*aconitate intermediate,^2^ as part of the second step of the Krebs cycle. Recessive pathogenic variants in *ACO2* were initially identified in the infantile cerebellar retinal degeneration syndrome (ICRD, MIM**#**614559), a severe early-onset autosomal recessive neurodegenerative condition with seizures and profound psychomotor retardation related to progressive cerebral and cerebellar degeneration, and ophthalmologic abnormalities involving optic nerve and retinal degeneration.^3–7^ Additional clinical presentations related to recessive *ACO2* variants were subsequently reported in individuals with a neuromuscular condition^8^^,^^9^ and syndromic presentations of hereditary spastic paraplegia^8^^,^^10^^,^^11^ (Table 1). Most of these patients presented with optic atrophy, and this predilection for optic nerve involvement is further highlighted by the identification of recessive *ACO2* mutations in seven individuals from four independent families that developed isolated optic atrophy (OPA9; MIM#616289).^6^^,^^12–14^

**Table 1:** Pathologies associated to *ACO2* dominant and recessive variants, with the number of individuals (ind) and families (fam) referenced

Pathology	Transmission	Number of cases reported	References
ICRD	Recessive	8 ind from 2 fam	Spiegel et al.^5^
		3 ind from 2 fam	Metodiev et al.^6^
		1 ind	Srivastava et al.^7^
		9 ind from 3 fam	Sharkia et al.^4^
		1 ind	Blackburn et al.^8^
Neuromuscular	Recessive	1 ind	Sadat et al.^9^
		1 ind	Blackburn et al.^8^
HSP ‘+’	Recessive	1 ind	Marelli et al.^11^
		11 ind	Bouwkamp et al.^10^
		1 ind	Blackburn et al.^8^
Ataxia, dysarthria, dev. delay	Recessive	2 ind from 1 fam	Blackburn et al.^8^
**ROA**	Recessive	**12 ind from 11 fam**	**This work**
		2 ind from 1 fam	Metodiev et al.^6^
		2 ind from 1 fam	Kelman et al.^12^
		1 ind	Chen et al.^13^
		2 ind from 1 fam	Gibson et al.^14^
**DOA**	**Dominant**	**66 ind from 50 fam**	**This work**

Inherited optic neuropathies (IONs) are an important cause of blindness in children and young adults. The pathological hallmark is the degeneration of the retinal ganglion cells (RGCs), whose axons converge to form the optic nerve, allowing the fast transmission of visual information from the retina to the brain. IONs are mainly transmitted as a dominant trait as in the case of Kjer disease or dominant optic atrophy (DOA). In the majority of cases, DOA is caused by *OPA1* mutations,^15^ and more rarely by pathogenic variants in additional genes involved in mitochondrial dynamics, including *MFN2*, *DNM1L*, *OPA3*, *AFG3L2* and *SPG7*,^16–21^ or in *SSBP1*, a gene involved in mtDNA replication.^22–24^ Recessive ION has also been identified in few individuals with isolated recessive optic atrophy (ROA), due to biallelic mutations in genes involved in the respiratory complex I assembly and activity, including *RTN4IP1*, *TMEM126A* and *NDUFS2*,^18^^,^^25^^,^^26^ and in *WFS1*, a gene involved in calcium transfer and interorganellar interactions between the endoplasmic reticulum and mitochondria.^27^ In both DOA and ROA cohorts, 10–20% of individuals with optic atrophy present additional extraocular features, such as sensorineural deafness, peripheral neuropathy, encephalopathies, movement disorders and myopathies.^28^^,^^29^

About 40% of individuals with IONs still do not have a confirmed molecular diagnosis,^30^ which prompted us to screen the *ACO2* gene in multicentre patient cohorts to determine its contribution to the overall disease burden. Here, we report the identification of 50 index cases carrying heterozygous pathogenic *ACO2* variants, together with 11 index cases with biallelic *ACO2* mutations. *In vitro* and *in silico* analyses of identified variants confirmed pathogenicity and deleterious effects on the first steps of the tricarboxylic citrate acid cycle and on the maintenance of the mitochondrial genome.

## Materials and methods

### Consent for genetic investigations

Written informed consent was obtained from each subject involved in this study or from the parents of subjects under 18 years of age, according to protocols approved by the different institutions involved in this study, and in agreement with the Declaration of Helsinki.

### Nomenclature

*ACO2* variants are described according to the NCBI transcript reference sequence NM_001098.2, including 18 exons, encoding a protein of 780 amino acids (reference sequence NP_001089.1). The numbering of the nucleotides reflects that of the cDNA, as recommended by the version 2.0 nomenclature of the Human Genome Variation Society (HGVS): http://varnomen.hgvs.org.^31^

### Molecular genetic analysis

After extraction of genomic DNA from peripheral blood cells, *ACO2* mutations were screened using resequencing gene panels dedicated to the molecular diagnosis of IONs or of mitochondrial inherited diseases. Variants were analysed by applying various prioritization filters described elsewhere.^21^^,^^32^ All dominant variants were absent or had minor allele frequency (MAF) threshold of <0.0001 in the Genome Aggregation Database (gnomAD v.2.1.1, https://gnomad.broadinstitute.org), while recessive variants were considered with an MAF threshold <0.005. All candidate pathogenic variants were validated by Sanger sequencing, and their segregation assessed in DNA from relatives, when available.

### *In silico* analysis of ACO2 missense mutations

The pathogenicity of *ACO2* missense variants was assessed using the SIFT,^33^ Polyphen-2^34^ and Mutation Taster^35^ prediction tools, and splice-site mutations were analysed by the dbscSNV programme. All variants were analysed using ACMG rules and classification (https://varsome.com/) (Supplementary Table 1). The impact of *ACO2* missense variants on the protein structure and function was determined first, by comparing the three-dimensional (3D) structure of the native and mutated ACO2 proteins,^36^ second, by performing the molecular dynamics simulation using Gromacs 5.1.4 software to determine the impact of variants on protein stability, amino acid flexibility and global protein dimension^37^ and third, by performing a molecular docking analysis using Autodock 4.2 to identify the impact of variants on the interaction between ACO2- [4Fe, 4S] (SF4) clusters and the interaction of the complex [ACO2-SF4] with the citrate, *cis-*aconitate and isocitrate ligands.^38^

### Fibroblast analysis

Fibroblasts from the dominant *ACO2* P15 patient (c.1253-1254insA) and the recessive P51 patient (c.36 + 5del; c.719G>C), as well as from an ICRD patient with biallelic *ACO2* variants (c.487G>A; c.2048G>A), were generated from skin biopsies and compared throughout the study to two wild-type fibroblast cell lines. Cells were cultivated in 2/3 Dulbecco’s Minimum Essential Medium (DMEM, Gibco) supplemented with 1/3 AmnioMAX (Gibco), 10% foetal calf serum (Lonza) and 1% Penicillin-Streptomycin-Amphotericin B (Lonza).

### Western blotting

Cellular proteins (20 μg) solubilized in Laemmli buffer were resolved by SDS-polyacrylamide gel electrophoresis and transferred to polyvinylidene difluoride membranes (GE Healthcare). For immune-detection, an anti-ACO2 monoclonal antibody (#ab110321, Abcam, 1/1000) was used. Monoclonal anti-VDAC and anti-α-tubulin antibodies were used as mitochondrial and cytosolic reference markers, respectively (Supplementary Fig. 1).

### Mitochondrial respiration rates

Mitochondrial oxygen consumption measurements were performed at 37°C and atmospheric pressure using a high-resolution oxygraph (O2K, Oroboros Instrument, Innsbruck, Austria).

Respiration rates on permeabilized cells were measured in respiratory buffer RB (10 mM KH_2_PO_4_, 300 mM mannitol, 10 mM KCl, 5 mM MgCl_2_, 0.5 mM EGTA and 1 mg/ml bovine serum albumin, pH 7.2) using different tricarboxylic citric acid cycle intermediates and different substrates of CI, CI+CII and CII as followed: First, state 2 (non-phosphorylating) respiration was measured after adding 5 mM citrate. Following the stimulation of the phosphorylating respiration by saturating ADP concentration (1.5 mM), 2.5 mM pyruvate was added, followed by 5 mM malate. Finally, 5 mM glutamate was added to check whether the CI-linked maximal phosphorylating respiration was reached. Succinate (10 mM) was then added to measure the combined CI and CII-linked respiration with convergent CI + II electron flow into the Q-junction corresponding to the maximal stimulated phosphorylating respiration (OXPHOS capacity). Rotenone (5 µM) was used to inhibit CI activity and thus to obtain the maximal CII-linked respiration. Oligomycin (F0F1-ATP synthase inhibitor, 4 µg/ml) and 1 µM of the mitochondrial uncoupler carbonyl cyanide p-trifluoromethoxyphenylhydrazone were sequentially added to ensure that the cells were fully permeabilized. Finally, antimycin A addition (2 µg/ml) was used to check for the non-mitochondrial oxidation.

### Enzymatic measurements

The activities of aconitase 2, fumarase and citrate synthase (CS) were measured at 37°C with an UV*mc*^2^ spectrophotometer (SAFAS, Monaco) on the mitochondrial enriched fraction. Cells were re-suspended in cell buffer [250 mM saccharose, 20 mM tris(hydroxymethyl)aminomethane, 2 mM EGTA, 1 mg/ml bovine serum albumin, pH 7.2; 50 μl/10^6^ cells], disrupted by two freezing-thawing cycles, washed, centrifuged for 1 min at 16 000 g to eliminate the cytosolic fraction and re-suspended in the cell buffer (125 μl/10^6^ cells). Aconitase activity was immediately assayed according to^39^ in Tris-HCl buffer (50 mM, pH 7.4) supplemented with 0.5 mM MnAcetate and containing 0.1% Triton-X100. One hundred millimolar isocitrate were used to initiate the reaction and *cis-*aconitate production was followed at 240 nm. Fumarase activity was measured in KH_2_PO_4_ buffer (50 mM, pH 7.4) supplemented with 0.1 mM EDTA and containing 0.1% Triton-X100. Ten millimolar malate was used to initiate the reaction and fumarate production was followed at 250 nm. CS activity was assayed by a standard procedure.^40^ The protein content was determined with the bicinchoninic assay kit (Uptima, Interchim, Montluçon, France) using bovine serum albumin as standard. Aconitase 2 activity was normalized to fumarase and CS as mitochondrial content markers. Enzymatic measurements were performed at least twice in duplicate, on two different cell pellets from different passages.

### Quantification of mtDNA copy number

For mtDNA quantification, total DNA was isolated from fibroblasts by using a High Pure PCR Template Preparation Kit (Roche ref 11796828001). Q-PCRs were performed in triplicate in 96-well reaction plates (Applied Biosystems). Each reaction (final volume 10 µl) contained 25 ng DNA, 5 µl of Power SYBR-Green PCR Master Mix (Applied Biosystems) and 0.5 µM of each forward and reverse primer. *COX1* and *MT*-*ND4*, mitochondrial encoded genes, were amplified and β2 microglobulin (β2 m) and *GAPDH* nuclear-encoded genes were used as a normalizing control. Primers were COX1: F-5′-TCCACTATGTCCTATCAATA-3′ and R-5′-GGTGTAGCCTGAGAATAG-3′; ND4: F-5′-CGCACTAATTTACACTCA-3′ and R-5′-GCTAGTCATATTAAGTTGTTG-3′; β2m: F-5′-CAGCTCTAACATGATAACC-3′ and R-5′-CCTGTAGGATTCTTCTTTC-3′. GAPDH: F-5′-CCCTGTCCAGTTAATTTC-3′ and R-5′-CACCCTTTAGGGAGAAAA-3′.

### Statistical analyses

**Figure 1:**
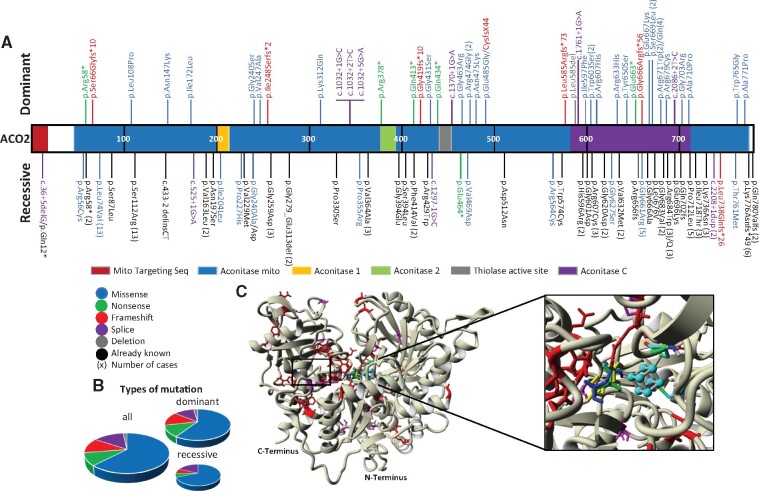
**Localization and quantification of the *ACO2* variants on the protein primary structure.** (**A**) The structure of the ACO2 protein including the different functional domains is represented, with all the dominant mutations identified shown on top, and all the recessive mutations identified shown below (different colours according to the type of variant), interspersed with the published recessive variants (in black). The number of times that a variant has been identified is indicated between brackets. (**B**) Representation of the different variant subtypes affecting *ACO2* in all optic atrophy cases (*left*), in dominant cases (*right top*) and in recessive cases (*right bottom*). There are no significant differences (*P*-value: 0.80) between dominant and recessive groups. (**C**) Three-dimensional structure of ACO2 protein showing the dominant (red) and recessive (magenta) variants and the ligands localization. [4Fe, 4S] cluster (cyan), citrate (green), *cis-*aconitate (blue) and isocitrate (yellow).

**Figure 2:**
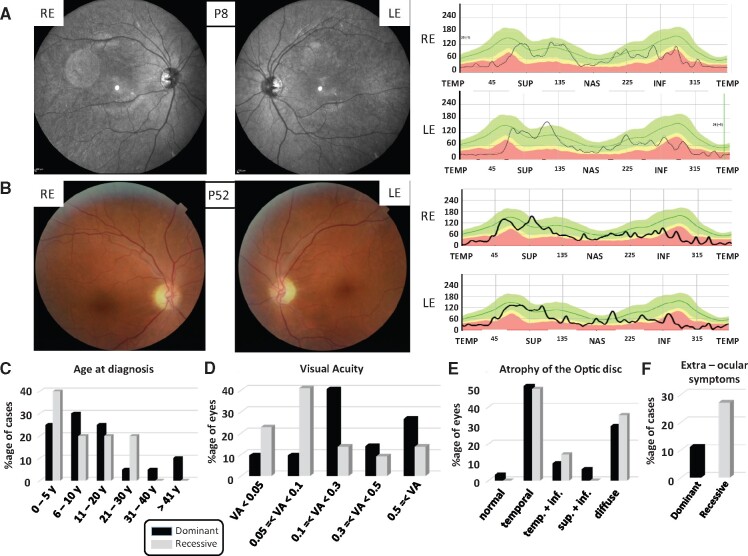
**Clinical data of the dominant and recessive *ACO2* individuals.** (**A** and **B**) *Left*: Eye fundus pictures of individuals with a dominant (**A**: P8) and a recessive (**B**: P52) *ACO2* mutation revealing the temporal pallor of the optic discs in both REs and LEs. *Right*: Evaluation of the retinal nerve fibre layer by optic coherence tomography in the same individuals, disclosing in both cases the retinal nerve fibre layer reduction in the temporal quadrants. The green area defines the 5th to 95th, the yellow area the 1st to 5th and the red area below the 1st percentiles. INF = inferior quadrants; LE = left eye; NAS = nasal; RE = right eye; SUP = superior; TEMP = temporal. (**C**) Ages at diagnosis categorized in six groups and represented as percentages of the number of individuals in the dominant and recessive cohorts (y = years) are not significantly different (*P*-value: 0.54). (**D**) Visual acuities (VA), categorized in five ranges, and represented as percentages of the total number of eyes are significantly different between dominant and recessive cases (*P*-value: 0.001). (**E**) Alterations of the optic disc, according to the different retinal nerve fibre layer quadrants were assessed by optical coherence tomography. Data are represented as percentages of each alteration from all eyes for which the data were available. (temp. = temporal; inf. = inferior; sup. = superior) and are not significantly different between dominant and recessive cases (*P*-value: 0.88). (**F**) Percentage of dominant and recessive *ACO2* individuals presenting extra-ocular symptoms.

Molecular data concerning the mutation types (Fig. 1) and clinical data, including age at diagnosis, visual acuity and segment of atrophy of the optic nerve (Fig. 2) were statistically compared between the dominant and recessive cases, using the Fisher exact test.

Biochemical data generated from the analysis of the mitochondrial respiration parameters, mtDNA amounts and protein abundancies were statistically compared between the dominant, recessive, ICRD and control samples, using the two-tailed paired *t*-test.

All data from this work are available upon request.

## Results

### Identification of ACO2 variants in individuals with ION

Cohorts including about 1000 molecularly undiagnosed ION individuals from France, Germany, Italy, Spain, Belgium and the UK were screened by targeted sequencing for the presence of *ACO2* variants. Among individuals with a single *ACO2* variant, we selected cases with an MAF lower than 0.0001, whereas in individuals with biallelic heterozygous composite or homozygous variants, we selected the cases with an MAF lower than 0.005 for each variant. This led to the identification of 50 individuals harbouring 1 of 43 different heterozygous mutations, among which 29 were novel, as well as 11 individuals with biallelic mutations, among which 8 were novel. A compilation of these variants and the associated amino acid changes are displayed on ACO2 primary sequence and 3D structure in Fig. 1A and C and listed in the Supplementary Table 1. Notably, four dominant variants p.(Arg474Gly), p.(Trp603Ser), p.(Ser669Leu) and p.(Arg671Trp) were identified in two unrelated individuals and p.(Arg671Gln) in four unrelated individuals, while one recessive variant, p.(Leu74Val), with an MAF value of 0.00369, was found in five unrelated ROA individuals, and also formerly described in eight individuals with syndromic *ACO2* presentations. This p.(Leu74Val) variant was associated once with p.(Gly661Arg) in the P56 individual, a combination already reported in an ROA family.^6^ Segregation analysis was performed where available. In DOA cases, all affected relatives carried the variant, whereas some relatives in the families of patients P6, P8, P9, P13 and P33 carrying the variant were asymptomatic, as occasionally observed in families with *OPA1* mutations.^15^^,^^41^ Moreover, segregation analysis revealed *de novo* mutations in the P15 and P18 index cases.

Analysis of the types of mutations identified in *ACO2* (Fig. 1B) revealed that two-third of all variants were missense mutations, irrespective of the dominant or recessive mode of inheritance, while nonsense, frame-shift, splice-site mutations and deletions accounted together for the third part of all mutations identified, without significant difference between the distribution of the mutation types between dominant and recessive cases (*P*-value: 0.80). Notably, the identification of null alleles in dominant cases suggests that *ACO2* haploinsufficiency contributes mainly to DOA pathophysiology.

### Clinical examination of individuals with ACO2 mutations

All individuals harbouring *ACO2* mutations had an ophthalmological examination. Optic atrophy was identified by fundus examination and confirmed by optical coherence tomography, which showed decreased retinal nerve fibre layer thickness (Fig. 2A and B). Additional ophthalmological features are summarized in Supplementary Table 1 and Fig. 2C. Age at first diagnosis of optic atrophy symptoms occurred essentially during the first two decades, without significant difference between dominant and recessive cases (*P*-value: 0.54; Fig. 2C). The distribution of the visual acuity from all eyes revealed that the recessive cases were significantly (*P*-value: 0.001) more severely affected than the dominant ones, with more than 60% of eyes from the ROA group having a visual acuity lower than 0.1, whereas more than 80% of eyes from the DOA group have a visual acuity above 0.1 (Fig. 2D). Analysis of the optic disc by optical coherence tomography and retinal nerve fibre layer thickness measurements indicate a preferential involvement of the temporal quadrant in both the dominant and recessive patient groups (Fig. 2E), suggesting a similar pattern of RGC degeneration, irrespective of the mode of transmission (*P*-value: 0.88). Assessment of colour vision revealed highly divergent alterations, including protan, deutan and tritan types of dyschromatopsy (data not shown). Notably, eight patients with DOA or ROA showed additional retinal alterations, including macular microcysts and a macular dystrophy in one case. In 6/50 (12%) of the dominant cases and 3/11 (27%) of the recessive cases, additional extraocular symptoms were found in addition to optic atrophy, with a single case showing hearing loss (P38) and two cases showing a late-onset cerebellar ataxia (P19 and P53).

### Evaluating the pathogenicity of ACO2 variants

*In silico* analysis was performed in order to ascertain the pathogenicity of identified *ACO2* variants. Each variant was evaluated by using Sift, Polyphen-2 and Mutation Taster prediction tools (Supplementary Table 1). All dominant variants, with the exception of the c.1454A>G [p.(Glu485Gly), P23] and c.1898G>A [p.(Arg633His), P32], were predicted to be either deleterious or disease causing by all three software. Similarly, the ACMG classification tool predicted that 19 were pathogenic (class 5), 10 likely pathogenic (class 4) and 13 with uncertain significance (class 3). Prediction results were less clear-cut regarding the recessive variants, with five classified as pathogenic (class 5), six likely pathogenic (class 4) and five with uncertain significance (class 3), suggesting that they might have milder effect on the protein activity. The seven variants affecting splice-sites were predicted to be deleterious by the dbscSNV tool, while only five affecting the ±1 or ±2 splicing position were classified as pathogenic, and two affecting the +5 position as of uncertain significance according to the ACMG rules (Supplementary Table 1).

To gain further insights on the effects of the missense variants on the ACO2 biophysical properties, we analysed each variant *in silico* using three sets of predictive tools. To infer the 3D structure of the human ACO2 protein, the 780 amino acids of the human sequence were aligned and positioned on the 3D crystal structure of the *Sus scrofa* ACO2 protein^42^ using the SwissModel server. Then, the ACO2 3D structure was modified with the PyMOL software to visualize all mutated proteins and minimized using the GROMACS software. Comparison of native and mutated ACO2 proteins showed that all variants affect the 3D structure by acquiring and/or losing hydrogen bonds and/or hydrophobic interactions, with the exception of the dominant p.(Gly240Ser) and p.(Gly431Ser), and the recessive p.(Gly240Ala) and p.(Val761Met) variants. Results of the molecular dynamic simulation revealed that all variants increased protein stability (RMSD), with the exception of the dominant p.(Val247Ala) variant, which decreased protein stability, and the dominant p.(Ile172Leu) and the recessive p.(Arg56Cys) variants, which displayed no change in protein stability. All variants were affected for the amino acid flexibility parameter (RMSF), and all had decreased protein dimension (Rg) except the dominant p.(Ile172Leu) and p.(Val247Ala) variants and the recessive p.(Arg56Cys) variants, which displayed a normal protein dimension. Molecular docking analysis of the native and mutated proteins complexed to the iron-sulphur cluster [ACO2-SF4] did not evidence a significant difference of their binding energy (−3.43 kcal/mol for the native ACO2 and from −3.81 to −3.38 kcal/mol for mutated proteins). All variants had also normal binding energies for citrate, isocitrate and *cis-*aconitate, with the exception of the dominant p.(Ser669Leu), p.(Arg671Gln), p.(Arg671Trp), p.(Gly703Arg) and p.(Ala710Pro) variants that displayed increased binding energy and/or alteration of the substrate binding to the SF4 complex (see Supplementary Table 2). Altogether, these *in silico* analyses reveal that all *ACO2* variants display altered biophysical properties, although our results did not identify a single criterion discriminating dominant from recessive mutations.

### *ACO2 mutated fibroblasts show reduced* ACO2 *abundance and activity, with alterations in the first steps of the Krebs cycle and depletion of mtDNA copy number*

**Figure 3:**
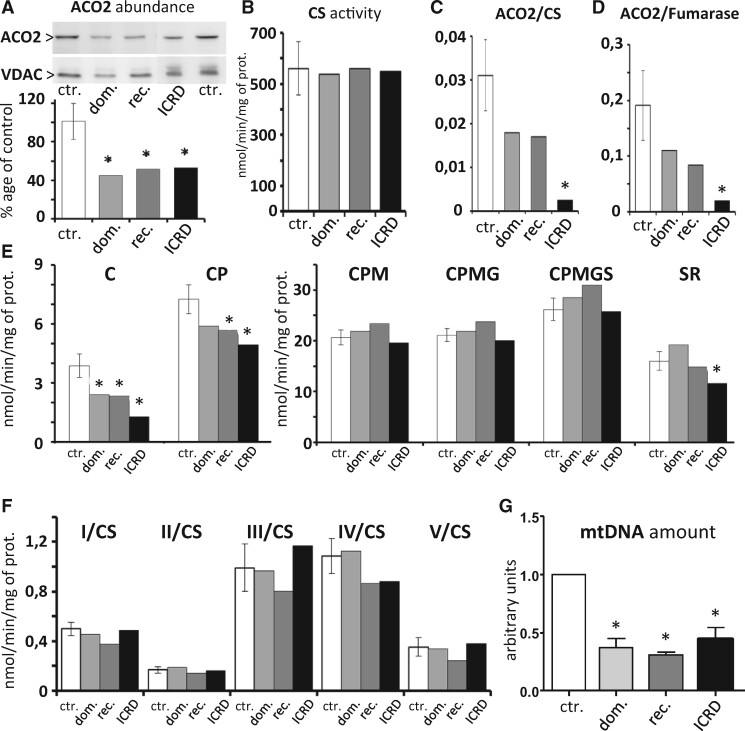
**Analysis of dominant and recessive *ACO2* mutated fibroblasts, compared to an *ACO2*-related ICRD fibroblast cell line.** All experiments were performed at least in two independent replicates for each control (ctr.) and dominant (dom.) and recessive (rec.) *ACO2* fibroblasts, and compared to one *ACO2*-related ICRD fibroblast cell line (ICRD). Results are mean ± SD. Statistical analysis of results from all the following experiments was performed using the two-tailed paired *t*-test. (**A**) Western blots with antibodies against ACO2 and VDAC proteins. The quantification of the relative ratio shows a significant decrease of ACO2 protein in all patient fibroblasts (*P-value <0.05). (**B**) CS activity is not affected by *ACO2* mutations. (**C** and **D**) Relative ACO2 activity normalized to the CS (C) and to the fumarase (D) activities shows a tendency to decrease in the dominant and recessive *ACO2* fibroblasts, and a significant decrease in the ICRD fibroblasts (*P-value <0.05). (**E**) The assessment of fibroblast respiration (mitochondrial oxygen rates related to maximal phosphorylation condition in permeablized fibroblasts) by oxygraphy, using the Krebs cycle substrates, Citrate (C), pyruvate (P), malate (M), glutamate (G), succinate (S), followed by the inhibition of complex I by rotenone (R), show that the respiration related to the use of citrate is decreased in all *ACO2* fibroblasts, partially increased by pyruvate, and fully restored by malate. Further stimulation by glutamate and succinate is limited, and only the ICRD fibroblasts are significantly more affected than the other fibroblasts by the Rotenone (*P-value <0.05). (**F**) Enzymatic activities from four independent experiments of the respiratory complexes (CI to CV) from the control and the *ACO2* mutated fibroblast strains related to the CS enzymatic activity did not reveal a significant difference between control and mutated fibroblasts. (**G**) Mitochondrial DNA copy number in *ACO2* mutated fibroblasts normalized to control fibroblasts reveals a significant decrease in the mitochondrial genome in all *ACO2* cells (*P-value <0.05).

In order to analyse the effects of dominant and recessive *ACO2* variants on the mitochondrial physiology, we generated skin fibroblasts from a DOA (P15) and an ROA (P51) patient, as well as from an ICRD patient carrying the c.487G>A/p.(Val163Met) and c.2048G>A/p.(Gly683Asn) variants. All cells displayed a normal growth in standard culture media, compared to controls (not shown). Western blot analysis revealed that ACO2 amounts were similarly reduced by half in the three mutated *ACO2* cell lines (Fig. 3A), while the CS remained unchanged, witnessing a regular amount of mitochondria in all cell lines (Fig. 3B). Assessment of the mitochondrial aconitase activity, relatively to the CS or the fumarase activity, disclosed a 50% decrease in both dominant and recessive backgrounds, whereas the activity was drastically reduced in the ICRD cell line (Fig. 3C and D). Assessment of mitochondrial oxygen consumption in the maximal phosphorylation condition from permeabilized fibroblasts, in the presence of citrate as the sole substrate evidenced a significant reduction of the respiration rate in the three mutated cell lines, which was more severe in the ICRD cell line. Addition of pyruvate stimulated the respiration by the production of one NADH related to the pyruvate to acetyl-coA reaction, but to a lower level in all *ACO2* cell lines compared to wild-type cells, with values remaining significant for the recessive *ACO2* and ICRD cells (Fig. 3E). Further addition of malate restored the respiration up to the level of the wild-type cell lines, and the subsequent CI-linked respiration with maximal substrate supply (pyruvate, malate, glutamate), or CI + CII-linked respiration (successive addition of succinate) were maintained to levels comparable to the wild-type cell lines, suggesting that an adaptation process by-passes the Krebs cycle bottleneck due to the reduced ACO2 activity. Finally, the CII-linked respiration did not significantly differ between the DOA and ROA cell lines, compared to controls, but was decreased for the ICRD cell line (Fig. 3E), witnessing a normal FADH2 production and complex II activity in both DOA and ROA. Further evaluation of the enzymatic activities of the respiratory complexes I to V did not reveal any significant difference in comparison with controls, irrespective of the *ACO2* genotype (Fig. 3F). Measurements of mtDNA abundance in the three *ACO2* mutated cell lines disclosed a significant reduction of more than 50% in the three mutated cell lines compared to the control cells (Fig. 3G), as already reported for cells lacking the mitochondrial aconitase activity.^9^ Further evaluation of the mitochondrial network shape did not reveal modification of the fusion–fission ratio in the dominant and recessive *ACO2* cells (data not shown).

Altogether, these data indicate that the *ACO2* dominant and recessive mutations affect significantly the mitochondrial aconitase activity and consequently the first steps of the Krebs cycle and the pyruvate/citrate dependent respiration, with a further depletion of the mitochondrial genome. Nevertheless, they also indicate that the mitochondrial respiration based on alternative Krebs cycle substrates can be restored to a normal level, by circumventing the metabolic bottleneck due to ACO2 deficiency in both DOA and ROA genetic backgrounds.

## Discussion

IONs are genetically heterogeneous, and reaching a confirmed molecular diagnosis can be challenging. In order to improve diagnostic pathways, we have screened large cohorts of European individuals with molecularly undiagnosed optic neuropathy to determine whether *ACO2* variants account for a proportion of cases. In addition to the seven ION cases that were found to harbour recessive *ACO2* mutations as previously described,^6^^,^^12–14^ this study highlights, for the first time, the important contribution of mono-allelic *ACO2* variants as a cause of isolated optic atrophy, with 50 independent cases carrying dominant pathogenic variants. Clearly, both dominant and recessive mutations in *ACO2* can cause the very same ophthalmological presentation with a pathological predilection for RGCs within the inner retina. Analyses of the clinical data indicate that *ACO2* mutations result in ophthalmological and optical coherence tomography features similar to those seen in the context of *OPA1* mutations,^15^^,^^43^ with the preferential involvement of the papillomacular bundle. There was variable disease severity with visual acuity ranging from normal in clinically asymptomatic individuals to severe visual impairment meeting the criteria for legal blindness. The age at which the diagnosis was first made mainly occurred during the first two decades of life in both dominant and recessive individuals. Notably, in 8 out of the 61 individuals with *ACO2* mutations mild retinal alterations, including macular microcysts and macular dystrophy, were noticed. While macular microcysts are a non-specific retinal alteration, possibly of mechanical origin, also reported in severely affected DOA and Leber Hereditary Optic Neuropathy cases,^44^^,^^45^ macular dystrophy, a truly degenerative process, converges with the retinal features described in ICRD and other syndromic clinical presentations related to *ACO2* recessive variants.^6^^,^^10^^,^^46^ In addition, 12% of dominant and 27% of recessive cases displayed extraocular symptoms that can either be incidentally related to the *ACO2* individuals, or as in *OPA1* cases, related to highly specific mutations. This could also be the consequence of ageing with an *ACO2* mutated genetic background, as individuals P19 aged 73 and P53 aged 61 both showed cerebellar related symptoms, mirroring a late-onset ICRD condition. Thus, although the *ACO2* dominant and recessive presentations that we identified are clinically similar, disclosing in most cases an isolated optic atrophy, the recessive cases do appear more severely affected than the dominant ones, suggesting that the combination of bi-allelic *ACO2* variants compromise to a higher level the aconitase 2 functions.

Concerning the dominant cases, we have identified 43 variants, among which 30 have not been referenced (absence of rs number). Two-thirds of these variants are missense changes that are predicted to be pathogenic, with the exception of the p.(Glu485Gly) and p.(Arg633His) amino acid changes that were identified in individuals (P23 and P32, respectively) with an asymmetric optic atrophy. The remaining third of the variants are essentially nonsense, frame-shift and splice mutations, most probably behaving as null alleles. This suggests that haploinsufficiency in ACO2 activity is the basic pathophysiological mechanism that drives RGC loss and optic nerve degeneration secondary to *ACO2* mutations, similarly to what is observed in *OPA1* cases. This should prompt an in-depth ophthalmological examination of parents of the recessive *ACO2* patients carrying such null alleles.

Regarding the recessive cases, among the 11 individuals with bi-allelic *ACO2* mutations, we identified eight novel variants, including three missense, three splice-site, one frame-shift, and one nonsense mutations. We also identified five individuals harbouring the previously reported p.(Leu74Val) variant which is the disease-associated variant most frequently observed in population databases (MAF = 0.00369). The p.(Leu74Val) variant was found in combination with the p.(Gly661Arg) variant in one individual, a genotype previously described in an ROA patient.^6^ For the four other individuals, the p.(Leu74Val) variant was combined with splice site and nonsense mutations, therefore, suggesting that the p.(Leu74Val) amino acid change has a mild effect on ACO2 function. This also applies for the p.(Gly240Ala) variant in patient P51, since this allele is associated with a splice-site mutation identified in our ROA cohort. Otherwise, all other recessive individuals displayed a combination of missense mutations that were never reported in any other *ACO2* associated clinical phenotype. Notably, the *in silico* analysis of all recessive missense mutations using the Sift, PolyPhen-2 and Mutation Taster software disclosed a combination of one variant with predicted pathogenicity with a second variant of less clear-cut pathogenicity in compound heterozygous individuals. Conversely, the two homozygous variants p.(Arg56Cys) and p.(Pro227His), identified in patients P52 and P55, respectively, were predicted to be deleterious and likely pathogenic, requiring further analyses to decipher their respective effects on ACO2 functions.

Additional biophysical analyses of the changes in the structure, in the molecular dynamics and the protein–ligand interactions of the missense mutated proteins revealed that all mutated proteins were altered with at least one out of the set of three criteria investigated, being affected. Nevertheless, none of these criteria could discriminate between dominant and recessive variants. However, some dominant variants affecting the ACO2 catalytic site located between the 669 and 710 amino acid positions, showed a clear alteration of the energy binding with aconitase substrates, emphasizing their key role for the aconitase enzymatic activity.

The functional consequences of the dominant and recessive *ACO2* variants identified in our study were probed further in patient-derived skin fibroblasts. As already observed for ROA individuals with biallelic *ACO2* mutations,^5^^,^^46^ the aconitase abundance and activity were reduced by half both in recessive and dominant cell lines, suggesting a common pathophysiological mechanism related to *ACO2* mutations irrespective of the mode of inheritance. This had a significant consequence on the oxidative process when using citrate and pyruvate substrates, with the first steps of the Krebs cycle being significantly impaired by the reduced aconitase activity, thus leading to a metabolic bottleneck for the OXPHOS process. Nevertheless, the addition of substrates involved downstream of the ACO2 step, restored a normal OXPHOS respiration, suggesting that the production of NADH and FADH2 is compensated by the reactions occurring downstream of isocitrate production. This further suggests that the mitochondrial respiratory chain complexes are intact in these cells given that no alteration of the maximal enzymatic activity of complexes I to V was observed, irrespective of the *ACO2* genetic background. Finally, similarly to what was already reported in other models and cellular conditions,^9^ we observed a significant depletion of the mtDNA, reinforcing the role of ACO2, or of its substrates, in the maintenance of the mitochondrial genome. This in the context of fibroblasts apparently did not impinge directly on the OXPHOS setup, however, most probably may become relevant in high energy-dependent tissues and cell types, in particular in RGCs. In fact, the partial but consistent reduction in mtDNA copy number provides an interesting link to the pathophysiological mechanism recently discovered in the *SSBP1*-related DOA, for which the instability of the mitochondrial genome is the primary alteration responsible for the disease.^22^^,^^24^ Congruently, as for the SSBP1-mutant patients, from the clinical standpoint in addition to optic atrophy, some retinal symptoms may also occur, such as peculiar retinal dystrophic changes.^22–24^

In conclusion, our study highlights the importance of dominant and recessive *ACO2* mutations in patients with isolated or syndromic optic atrophy phenotypes. Dominant mutations are far more frequent than recessive ones at least in European populations, placing *ACO2* as the third most frequent genetic cause of IONs, after *OPA1* and *WFS1*. This also adds a new pathophysiological pathway involving mitochondria, as responsible for RGC degeneration, after the alterations of the mitochondrial dynamics in DOA and of the complex I assembly in ROA, connecting the first steps of the Krebs cycle to the OXPHOS respiration and the maintenance of the mitochondrial genome integrity. The next challenge is to investigate the underlying pathophysiological mechanisms in greater detail, to develop potential therapeutic interventions that will promote RGC survival in the presence of *ACO2* mutations.

## Supplementary material

Supplementary material is available at *Brain Communications* online.

## Funding

We are indebted to the financial support of the Université d’Angers, Centre Hospitalier Universitaire d’Angers, the Région Pays de la Loire, Angers Loire Métropole, the Fondation Maladies Rares, Fondation pour la Recherche Médicale, Retina France, Union Nationale des Aveugles et Déficients Visuels, Association Française contre les Myopathies, Ouvrir Les Yeux, Kjer-France, Association *ACO2* gène, Fondation de France and the Fondation VISIO. Parts of the work was funded by grants of the Deutsche Forschungsmeinschaft [Wi1189/11–1 to B.W.], and the Agence Nationale de la Recherche [17-RAR3-0007–01 to G.L.] as part of a joint research project ‘TreatOPON’ within the framework of the ERA-NET E-Rare 3 call. We thank the Italian Ministry of Health for financial support with the ‘ricerca corrente’ funding to V.C. and C.L.M., and the grant GR-2016–02361449 (IPHON project to L.C.). P.Y.-W.-M. is supported by a Clinician Scientist Fellowship Award (G1002570) from the Medical Research Council (UK), and also receives funding from Fight for Sight (UK), the Isaac Newton Trust (UK), the Addenbrooke’s Charitable Trust, the National Eye Research Centre (UK), the UK National Institute of Health Research (NIHR) as part of the Rare Diseases Translational Research Collaboration, and the NIHR Biomedical Research Centre based at Moorfields Eye Hospital National Health Service (NHS) Foundation Trust and University College Londo Institute of Ophthalmology. The views expressed are those of the author(s) and not necessarily those of the NHS, the NIHR or the Department of Health.

## Competing interests

The authors report no competing interests.

## Supplementary Material

fcab063_Supplementary_Data

## Appendix

The European ION Group is composed of the following collaborators: Francesca Bisulli, Catherine Vignal, Sabine Defoort-Dhellemmes, Isabelle Drumare Bouvet, Yaumara Perdomo Trujillo, Sylvie Odent, Helene Dollfus, Carmen Ayuso, Dan Milea, Astrid Plomp, Arturo Carta, Anna Maria De Negri, Costantino Schiavi, Maria Lucia Cascavilla, Piero Barboni, Klaus Rüther, Wolf A Lagrèze.

The affiliations of these collaborators can be found in the Supplementary Material.

## Abbreviations

3D: three-dimensional
CS: citrate synthase
DOA: dominant optic atrophy
ICRD: infantile cerebellar retinal degeneration syndrome
ION: inherited optic neuropathy
MAF: minor allele frequency
mtDNA: mitochondrial DNA
OXPHOS: oxidative phosphorylation
RGC: retinal ganglion cell
ROA: recessive optic atrophy
