# Supplementary material for: Dominant ACO2 mutations are a frequent cause of isolated optic atrophy
Source: Brain Commun. 2021 Apr 7;3(2):fcab063. doi: 10.1093/braincomms/fcab063 (PMC8152918; doi:10.1093/braincomms/fcab063)
Supplement: fcab063_Supplementary_Data [file fcab063_supplementary_data.pdf]

supplementary Table 1 FINAL

Pat #	Age	Sex	Age at diagnosis	BCVA	Visual field	Optic discs	Other symptoms	cDNA variant	protein change	Geno-type	Segregation	rs #	gnomAD frequency	Sift	PP2	MT	dbscSNV	ACMG criteria	ACMG classification
														Score					
**1**	25	F	unk.	OD: 0.5; OS: 0.63	NA	ODS: diffuse loss	none	c.172C>T	p.(Arg58*)	HT	no DNA	rs751460831	3,98E-06			1		PVS1	5
**2**	4	M	since birth	OD: 0.25; OS: 0.2	NA	ODS: temporal atrophy	none	c.196_197del	p.(Ser66Glyfs*10)	HT	grandfather affected; no DNA	unk.						PVS1	5
**3**	45	M	unk.	OD: 0.7; OS: 0.7	paracentral scotoma	NA	none	c.323T>C	p.(Leu108Pro)	HT	no DNA	unk.		0	1	1		PM2, PP2, PP3	3
**4**	8	M	since birth	OD: 0.4; OS: 0.13	OD: central scotoma, OS: paracentral scotoma	ODS: temporal atrophy	photophobia, manifest nystagmus latent to occlusion, OS esotropia	c.441C>G	p.(Asn147Lys)	HT	father affected (+/-); mother unaffected (+/+)	unk.		0,001	0.997	0.999		PS3, PM2, PP2, PP3	4
**5**	46	F	before 10 y	OD: 0.5; OS: 0.05	large centrocecal scotoma	ODS: temporal atrophy, uncapped papilla	left amblyopia	c.514A>C	p.(Ile172Leu)	HT	no DNA	unk.	4,08E-06	0,001	0.901	1		PS3, PM2, PP2, PP3	4
**6**	38	M	32 y	ODS: 0.1	ODS: central scotoma	acute loss of vision	head trauma at 10 y, followed by epilepsy; meningeal syndrome, diabetes insipidus since 2015; diffuse algebra syndrome; intolerance to the effort.	c.718G>A	p.(Gly240Ser)	HT	mother asymptomatic (+/-)	rs764138297	6.76E-05	0,001	0,993	1		PS3, PM2, PP2, PP3	4
**7**	50	M	unk.	OD:<0.05; OS: 0.9	NA	ODS: diffuse atrophy	none	c.740T>C	p.(Val247Ala)	HT	no DNA	rs750687436	3,98E-06	0	0,965	1		PS3, PM2, PP2, PP3	4
**8**	16	M	11 y	OD: 0.2; OS: 0.4	large annular central scotoma	ODS: temporal atrophy	none	c.741del	p.(Ile248Serfs*2)	HT	affected sister and mother (+/-); asymptomatic grandfather (+/-); unaffected father and grandmother (+/+)	unk.						PVS1	5
**9**	30	M	13 y	OD: 0.1; OS: 0.5	central scotoma	ODS: diffuse atrophy	macular dystrophy	c.935G>A	p.(Lys312Gln)	HT	father asymptomatic (+/-); mother unaffected (+/+)	rs1191153529		0	1	1		PM2, PP2, PP3	3
**10**	21	M	9 y	OD: 0.2; OS: 0.5	central scotoma	NA	none	c.1032+1G>C	p.?	HT	no DNA	unk.				1	0,9999	PVS1	5
**11**	30	M	19 y	ODS: 0.7	centrocecal scotoma	ODS: temporal atrophy	none	c.1032+2T>C	p.?	HT	no DNA	unk.				1	0,9999	PVS1	5
**12**	29	M	25 y	OD: 1; OS: 0.9	central scotoma	ODS: temporal atrophy	none	c.1032+5G>A	p.?	HT	father affected (+/-); mother unaffected (+/+)	unk.					0,9999	PM2	3
**13**	30	F	3 y	OD: 0.15; OS: 0.25	centrocecal scotoma	NA	none	c.1132C>T	p.(Arg378*)	HT	father asymptomatic (+/-); mother unaffected (+/+)	unk.						PVS1	5
**14**	16	M	13 y	OD: 0.3; OS: 0.2	NA	ODS: temporal atrophy	microcysts in macula	c.1237C>T	p.(Gln413*)	HT	no DNA	unk.						PVS1	5
**15**	21	F	9 y	ODS: 0.2	centrocecal scotoma	ODS: temporal and inferior atrophy	none	c.1254dup	p.(Gly419fs*10)	HT	unaffected parents (+/+)	unk.						PVS1	5
**16**	58	F	20 y	OD: 0.63; OS: 0.4	centrocecal scotoma	ODS: normal RNFL; small papilla poorly limited	strong myopia since childhood; cataract at 56 y	c.1291G>A	p.(Gly431Ser)	HT	no DNA	rs772704469	1,59E-05	0	0,994	1		PS3, PM1, PM2, PP2, PP3	5
**17**	33	M	15 y	OD: 0.2; OS: 0.1	central scotoma	ODS: diffuse atrophy	none	c.1300C>T	p.(Gln434*)	HT	brother unaffected (+/+)	rs1419587280	4,01E-06					PVS1	5
**18**	50	M	36 y	OD: 0.32; OS: 0.75	centrocecal scotoma OD>OS	ODS: temporal and inferior atrophy	none	c.1370+1G>A	p.?	HT	unaffected parents (+/+)	unk.				1	0,9999	PVS1	5
**19**	73	M	50 y	OD: 0.8; OS: 1	normal	normal	cerebelar ataxia	c.1387G>C	p.(Gly463Arg)	HT	no DNA	rs2006715	2,12E-05	0,004	1	1		PS3, PM2, PP2, PP3	4
**20**	64	F	42 y	ODS: 0.1	centrocecal scotoma	NA	none	c.1420A>G	p.(Arg474Gly)	HT	no DNA	unk.		0	1	1		PM2, PP2, PP3	3
**21**	36	F	3 y	OD: 0.1; OS: 0.1	central scotoma	ODS: diffuse atrophy	none				mother and brother affected (+/-)								
**22**	28	F	3 y	ODS: 0.1	central scotoma	ODS: diffuse atrophy; small optic disc; inferior hypoplasia	none	c.1425C>G	p.(Asn475Lys)	HT	affected son (+/-)	unk.		0	1	1		PM2, PP2, PP3	3
**23**	28	F	15 y	ODS: 0.1	central scotoma	ODS: diffuse atrophy	RPE dysomogeneity	c.1454A>G	p.(Glu485Gly)	HT	no DNA	unk.		0,455	0,001	0,999		PM2, PP2, BP4	3
**24**	29	F	since birth	OD: 0.032; OS: LP	OD: superonasal scotoma; OS: NA	ODS: temporal pallor; small disk	congenital nystagmus, exotropia OS, migraine	c.1452_1459del	p.(Glu485Cysfs*44)	HT	father affected (+/-); mother and 3 brothers/sisters unaffected (+/+)	unk.						PVS1	5
**25**	20	M	3 y	OD: 0.1; OS: 0.08	central scotoma	ODS: temporal atrophy	microcysts in macula	c.1753_1759del	p.(Leu585Argfs*73)	HT	no DNA	unk.						PVS1	5
**26**	16	M	before 10 y	OD: 0.08; OS: 0.1	central scotoma	ODS: diffuse atrophy	none	c.1753_1755delCTC	p.(Leu585del)	HT	father affected (+/-); mother unaffected (+/+)	unk.						PM2, PM4	3
**27**	36	M	6 y	OD: 0.063; OS: 0.063	paracentral scotoma	ODS: diffuse atrophy	none	c.1761+1G>A	p.?	HT	mother affected (+/-); father unaffected (+/+)	unk.					0,9999	PVS1	5
**28**	41	M	before 10 y	OD: 0.4; OS: 0.3	central scotoma	NA	none	c.1789A>T	p.(Ile597Phe)	HT	no DNA	unk.		0	1	1		PM2, PP2, PP3	3
**29**	8	M	4 y	OD: 0.08; OS: 0.15	central scotoma	ODS: superior and inferior atrophy	none	c.1808G>C	p.(Trp603Ser)	HT	no DNA	unk.		0	0,994	1		PS3, PM2, PP2, PP3	4
**30**	30	M	3 y	OD: 0.01; OS: 0.14	central scotoma	ODS: temporal atrophy	microcysts in macula; Barlow disease, normal MRI				no DNA								
**31**	38	M	unk.	ODS: 0.8	centrocecal scotoma	NA	none	c.1820G>A	p.(Arg607His)	HT	no DNA	rs1213893717	3,99E-06	0	1	1		PM2, PM5, PP2,PP3	4
**32**	56	F	54 y	OD: CF; OS: 1.0	NA	temporal gliosis	metabolic syndrome	c.1898G>A	p.(Arg633His)	HT	no DNA	rs371183037		0,017	0,004	0,999		PS3, PM2, PP2, PP3	4

**33**	9	M	8 y	OD: 0.8; OS: 1	NA	ODS: temporal atrophy	none	c.1949A>C	p.(Tyr650Ser)	HT	mother asymptomatic (+/-)	unk.		0	1	1		PM2, PM3, PP3	3
**34**	56	F	before 10 y	ODS: CF	central scotoma	NA	none	c.1987G>T	p.(Glu663*)	HT	no DNA	unk.						PVS1	5
**35**	26	F	4 y	OD: 0.1; OS: 0.12	ODS: centro + superior scotoma	ODS: superior and inferior atrophy	none	c.1995dup	p.(Gly666Argfs*46)	HT	no DNA	unk.						PVS1	5
**36**	33	F	6 y	ODS: 0.08	paracentral scotoma	NA	none	c.1999G>A	p.(Glu667Lys)	HT	no DNA	unk.		0	1	1		PM2, PP2, PP3	3
**37**	36	M	16 y	OD: 0.1; OS: 0.3	OS: central scotoma OD: NA	NA	none	c.2006C>T	p.(Ser669Leu)	HT	no DNA	rs764832926	4.02E-06	0	1	1		PM2, PP2, PP3	3
**38**	44	F	35 y	ODS: 0.32	paracentral scotoma	ODS: temporal atrophy	hearing loss				father affected with hearing loss								
**39**	71	M	63 y	OD: 0.5; OS: 1	centrocecal scotoma	ODS: superior and inferior atrophy	hypoacusis	c.2011C>T	p.(Arg671Trp)	HT	no DNA	rs377518755	4.01E-06	0	1	1		PS1, PS3, PM2, PP2,PP3	5
**40**	58	F	13 y	OD: 0.2; OS: 0.5	central scotoma	ODS: temporal atrophy	hypothyroidism				no DNA								
**41**	22	M	2 y	OS: 0.15; OD: 0.1	centrocecal scotoma	ODS: diffuse atrophy	none	c.2012G>A	p.(Arg671Gln)	HT	mother and grandfather affected (+/-); father and grandmother unaffected (+/+)	rs755024692	8.01E-06	0,002	1	1		PS1, PS3, PM2, PP2,PP3	5
**42**	32	M	7 y	OD: 0.2; OS: 0.16	central scotoma	ODS: temporal atrophy	none				mother affected (+/-); father unaffected (+/+)								
**43**	25	M	12 y	OD: 0.4; OS: 0.8	small centrocecal scotoma	ODS: temporal atrophy	ptosis OS				no DNA								
**44**	61	F	unk.	OD: 0.32; OS: 0.8	central scotoma	OD: diffuse atrophy, OS: temporal atrophy	small and dysmorphic optic disk; cataract and glaucoma OD				no DNA								
**45**	30	M	before 10 y	OD: 0.12; OS: 0.06	centrocecal scotoma	NA	macular dystrophy with reduced central multifocal ERG responses	c.2035C>T	p.(Arg679Cys)	HT	no DNA	rs747898560	4.01E-06	0	1	1		PM2, PP2, PP3	3
**46**	41	M	unk.	OD: 0.05; OS: 0.08	central scotoma	NA	none	c.2086+2T>C	p.?	HT	no DNA	unk.					0,9999	PVS1	5
**47**	43	M	14 y	OD: 0.17; OS: 0.16	paracentral scotoma	ODS: temporal atrophy	none	c.2107G>C	p.(Gly703Arg)	HT	mother affected (+/-); father unaffected (+/+)	unk.		0	1	1		PS3, PM2, PP2, PP3	4
**48**	5	M	5 y	OD: CF; OS: 0.05	central scotoma	ODS: diffuse atrophy	none	c.2128G>C	p.(Ala710Pro)	HT	no DNA	unk.		0,007	0,877	0,999		PS3, PM2, PP2, PP3	4
**49**	40	M	7 y	OD: 0.08; OS: 0.12	NA	NA	macula fanned bds; meningitis	c.2293T>G	p.(Trp765Gly)	HT	no DNA	unk.		0	1	1		PM2,PP2, PP3	3
**50**	15	F	unk.	ODS: 0.03	central scotoma	NA	none	c.2311G>C	p.(Ala771Pro)	HT	no DNA	unk.		0	1	1		PM2,PP2, PP3	3
**51**	30	M	4 y	ODS: <0.1	central scotoma	ODS: diffuse atrophy	epilepsy at 22; memory disorder and fatigability; normal MRI	c.36+5delG	p.?	HC	unaffected parents are HT for each variant	unk.					0,9999	PM2, PP3	3
								c.719G>C	p.(Gly240Ala)			rs141878785	0.000585	0,001	0,608	1		PS3, PM2, PP2, PP3	4
**52**	59	M	unk.	ODS: 0.05	centrocecal scotoma	ODS: temporal atrophy; uncapped papilla	none	c.166C>T	p.(Arg56Cys)	HM	brother (-/-); sister (+/-)	rs746995807	0	0	1	1		PS3, PM2, PP2, PP3	4
**53**	61	M	30 y	ODS: 0.1	NA	NA	kinetic and static cerebellar syndrome with cerebellar atrophy, episode of dysarthria at 45 y; migraine-related GCC, chronic diffuse pain and a cerebellar atrophy	c.220C>G	p.(Leu74Val)	HC	no DNA	rs141772938	0.0037	0,001	0,554	0,999		PP2, PP3, PP5	3
								c.1390G>T	p.(Glu464*)			unk.						PVS1	5
**54**	45	M	27 y	OD: CF; OS: 1.2	OD: large scotoma	NA	none	c.610A>C	p.(Ile204Leu)	HC	no DNA	rs774011999	3.19E-05	0,996	0,027	1		PS3, PM2, PP2	4
								c.1690C>T	p.(Arg564Cys)			rs781117483	7,96E-06	0	1	1		PS3, PM2, PP2, PP3	4
**55**	19	F	16 y	ODS: 0.05	NA	ODS: severe atrophy	strabismus in childhood; asymmetrical banal myopia	c.680C>A	p.(Pro227His)	HM	no DNA	unk.		0	1	1		PS3, PM2, PP2, PP3	4
**56**	53	M	2 y	OD: 0.015; OS: CF	NA		none	c.220C>G	p.(Leu74Val)	HC	no DNA	rs141772938	0.0037	0,001	0,554	0,999		PP2, PP3, PP5	3
								c.1981G>A	p.(Gly661Arg)			rs752034900	4,00E-06	0	1	1		PVS1,PM2, PP2, PP3, PP5	5
**57**	32	F	before 10 y	ODS: 0.25	central scotoma OD>OS	OS: diffuse atrophy; OD: temporal atrophy	intraretinic cysts	c.1064C>G	p.(Pro355Arg)	HC	parents are (+/-) and (-/+); unaffected sister (+/-)	unk.		0	1	1		PM2, PP2, PP3	3
								c.1879G>A	p.(Gly627Ser)			rs145042292	0.000368	0,022	0,06	1		PM2, PP2, PP3	3
**58**	21	M	11 y	OD: 0.8; OS: 0.4	OD: paracentral scotoma; OS: central scotoma	ODS: temporal and inferior atrophy	moderate photophobia	c.2282C>T	p.(Thr761Met)	HC	no DNA	rs142767544	4.4E-05	0,095	0,166	1		PM2, PP2, BP4	3
								c.1406T>A	p.(Val469Asp)			unk.		0	1	1		PS3, PM2, PP2, PP3	4
**59**	6	M	since birth	OD: 0.5; OS: 0.4	NA	ODS: temporal atrophy	none	c.220C>G	p.(Leu74Val)	HC	2 affected brothers; no DNA	rs141772938	0.0037	0,001	0,554	0,999		PP2, PP3, PP5	3
								c.2212_2213delCT	p.(Leu738Glnfs*26)			unk.						PVS1	5
**60**	7	M	6 y	OD: 0.25; OS: 0.08	central scotoma	ODS: temporal atrophy	none	c.220C>G	p.(Leu74Val)	HC	no DNA	rs141772938	0.0037	0,001	0,554	0,999		PP2, PP3, PP5	3
								c.525+1G>A	p.?			unk.					0,9999	PVS1	5

**61**	36	F	3 y	OD: LP; OS: HM	NA	NA	migraine; anorexia	c.220C>G	p.(Leu74Val)	HC	unaffected mother (+/-)	rs141772938	0.0037	0,001	0,554	0,999		PP2, PP3, PP5	3
								c.1297-1G>C	p.?			unk.					0,9999	PVS1	5

Supplementary Table 1: Clinical data of the 61 ACO2 individuals.

Legend: OD: oculus dexter; OS: oculus sinister; y: years; BCVA: best corrected visual acuity; CF: count fingers; HM: hand movement; LP: light perception; HT: heterozygous; HM: homozygous; HC: heterozygous composite; unk.: unknown; NA: not analyzed; "–": presence of the mutated allele; "+": presence of the wild type allele. ACMG classification: 5: pathogenic; 4: likely pathogenic; 3: uncertain significance.

supplementary Table 2 Final

	**Local structure changes (YASARA viewer)**					**Molecular dynamic simulation (Gromacs)**			**Protein-ligands interaction (Autodock 4.2)**				
Variant	HB losed (N°)	HI losed (N°)	HB gained (N°)	HI gained (N°)	Comments	Protein Stability (RMSD)	Amino acids Flexibility (RMSF)	Protein dimension (Rg)	Binding energy of [ACO2]-SF4 interaction (Kcal/mol)	Binding energy of [(ACO2-SF4)-citrate] (Kcal/mol)	Binding energy of [(ACO2-SF4)-cis aconitate] (Kcal/mol)	Binding energy of [(ACO2-SF4)-isocitrate]] (Kcal/mol)	**Comments**
**Native ACO2**									-3,43	-8,74	-7,83	-6,46	
**p.(Ile172Leu)**	_	1	_	2	change	no change	affected	no change	-3,38	-8,92	-7,32	-6,82	no change
**p.(Asn147Lys)**	2	_	_	1	change	increased	affected	decreased	-3,49	-8,44	-7,08	-6,19	no change
**p.(Gly240Ser)**	_	_	_	_	no change	increased	affected	decreased	-3,48	-8,58	-7,35	-6,53	no change
**p.(Val247Ala)**	_	2	_	_	change	decrease	affected	no change	-3,48	-8,57	-7,23	-6,2	no change
**p.(Gly431Ser)**	_	_	_	_	no change	increased	affected	decreased	-3,48	-8,53	-7,39	-6,23	no change
**p.(Gly463Arg)**	_	_	_	_	change	increased	affected	decreased	-3,42	-8,24	-7,75	-5,93	no change
**p.(Trp603Ser)**	_	5	1	_	change	increased	affected	decreased	-3,73	-8,47	-7,35	-6,11	no change
**p.(Arg633His)**	2	_	_	1	change	increased	affected	decreased	-3,69	-8,71	-7,2	-6,68	no change
**p.(Ser669Leu)**	1	_	_	_	change	increased	affected	decreased	-3,81	-4,7	-3,95	-3,24	binding energy increased and Isocitrate does not interact with SF4
**p.(Arg671Gln)**	4	1	1	_	change	increased	affected	decreased	-3,72	-6,6	-5,45	-4,11	binding energy increased
**p.(Arg671Trp)**	4	1	_	1	change	increased	affected	decreased	-3,8	-2,3	-2,34	-1,31	binding energy increased and all ligands do not interact with SF4

**p.(Gly703Arg)**	_	_	2		change	increased	affected	decreased	-3,61	-8,72	-8,04	-6,68	citrate does not interact wth SF4 cluster
**p.(Ala710Pro)**	_	_	_	1	change	increased	affected	decreased	-3,69	-8,27	-7,36	-6,7	citrate does not interact wth SF4 cluster
**p.(Arg56Cys)**	2	_	_	_	change	no change	affected	no change	-3,44	-8,69	-7,58	-6,04	no change
**p.(Leu74Val)**	2	_	_	1	change	increased	affected	decreased	-3,47	-8,63	-6,96	-6,85	no change
**p.(Ile204Leu)**	_	2	_		change	increased	affected	decreased	-3,46	-8,86	-7,87	-6,34	no change
**p.(Pro227His)**	1	_	_	1	change	increased	affected	decreased	-3,47	-8,78	-7,74	-6,73	no change
**p.(Gly240Ala)**	_	_	_	_	no change	increased	affected	decreased	-3,46	-8,44	-7,7	-5,64	no change
**p.(Val469Asp)**	_	2	_	_	change	increased	affected	decreased	-3,44	-8,78	-7,77	-6,6	no change
**p.(Arg564Cys)**	3	_	_	_	change	increased	affected	decreased	-3,69	-8,45	-6,87	-5,6	no change
**p.(Gly661Arg)**	_	_	1	3	change	increased	affected	decreased	-3,72	-8,73	-7,47	-6,18	no change
**p.(Gly683Asp)**	_	_	2	2	change	increased	affected	decreased	-3,45	-8,68	-6,79	-5,96	no change
**p.(Pro712Leu)**	_	_	_	1	change	increased	affected	decreased	-3,46	-8,64	-7,7	-6,77	no change
**p.(Thr761Met)**	_	_	_	_	no change	increased	affected	decreased	-3,73	-8,51	-7,73	-6,78	no change

Supplementary Table 2: Impact of missense variants on ACO2 protein structure, using the YASARA viewer, Gromacs 5.1.4 and Autodock 4.2 softwares.

Dominant variants are in black; recessive variants are in red. HB: hydrogen bond, HI: hydrophobic interaction, *: hydrogen bond.

Changes in ACO2 biophysical parameters were considered as fulfilling the PS3 criteria from the ACMG classification.

METHODS: In silico analysis of the ACO2 missense mutations

The FASTA sequence of ACO2 protein was retrieved from the UNIPROT database (Q99798) and the 3D structure of the native ACO2 protein was determined using the SWISS-MODEL servers (Waterhouse et al. 2018). The most suitable template to build the 3D model was selected on the basis of sequence identity and the QMEAN function.

The 3D mutant structures were generated using Pymol software, and the energy minimization for all 3D structures was done using the GROMACS 5.1.4

software. The 3D structure differences between the native and mutated ACO2 proteins were determined using the YASARA viewer software (Krieger et Vriend 2014).

The molecular dynamics simulations of the native and mutated ACO2 proteins were performed using the GROMACS 5.1.4 software package, with the force field CHARMM 27 (MacKerell et al., 1998). The production simulations were performed at 300K for 10 ns. Gmx rms, gmx rmsf and gmx gyrate commands were used to calculate the root-mean-square deviation (RMSD), the root-mean-square fluctuation (RMSF) and radius of gyration (Rg), respectively. All graphs were created using the QtGrace software.

AutoDock4.2 (ADT) was used for the molecular docking studies. The pdb files of the [4Fe,4S] cluster, citrate, cis-aconitate and isocitrate ligands were downloaded from the RCSB PDB protein data base (https://www.rcsb.org/), prepared using ADT and saved in PDBQT format. The native and mutated ACO2 structures were obtained using the SwissModel servers, prepared using ADT and saved in PDBQT format. The grid box size was set at 64, 54, and 62 Å for x, y, and z, respectively. The spacing between the grid points was 0.375 Å. The grid center was set at 38.067, 35.197 and 70.766 Å for x, y, and z, respectively. The Lamarckian Genetic Algorithm (LGA) was chosen to search for the best conformers, and all the docking processes were performed with the default parameters of AutoDock 4.2. All figures representing structures were produced using Ligplot+ with the default parameters.

supplemental references:

Krieger E and Vriend G.
YASARA View—molecular graphics for all devices—from smartphones to workstations. Bioinformatics 2014; 30 (20): 2981-82.

MacKerell AD, Bashford D, Bellott M, Dunbrack RL, Evanseck JD, Field MJ, et al.
All-atom empirical potential for molecular modeling and dynamics studies of proteins. J Phys Chem B. 1998 Apr 30;102(18):3586-616

Waterhouse A, Bertoni M, Bienert S, Studer G, Tauriello G, Gumienny R, et al.
SWISS-MODEL: homology modelling of protein structures and complexes.
Nucleic Acids Res. 2018 Jul 2;46(W1):W296-W303.

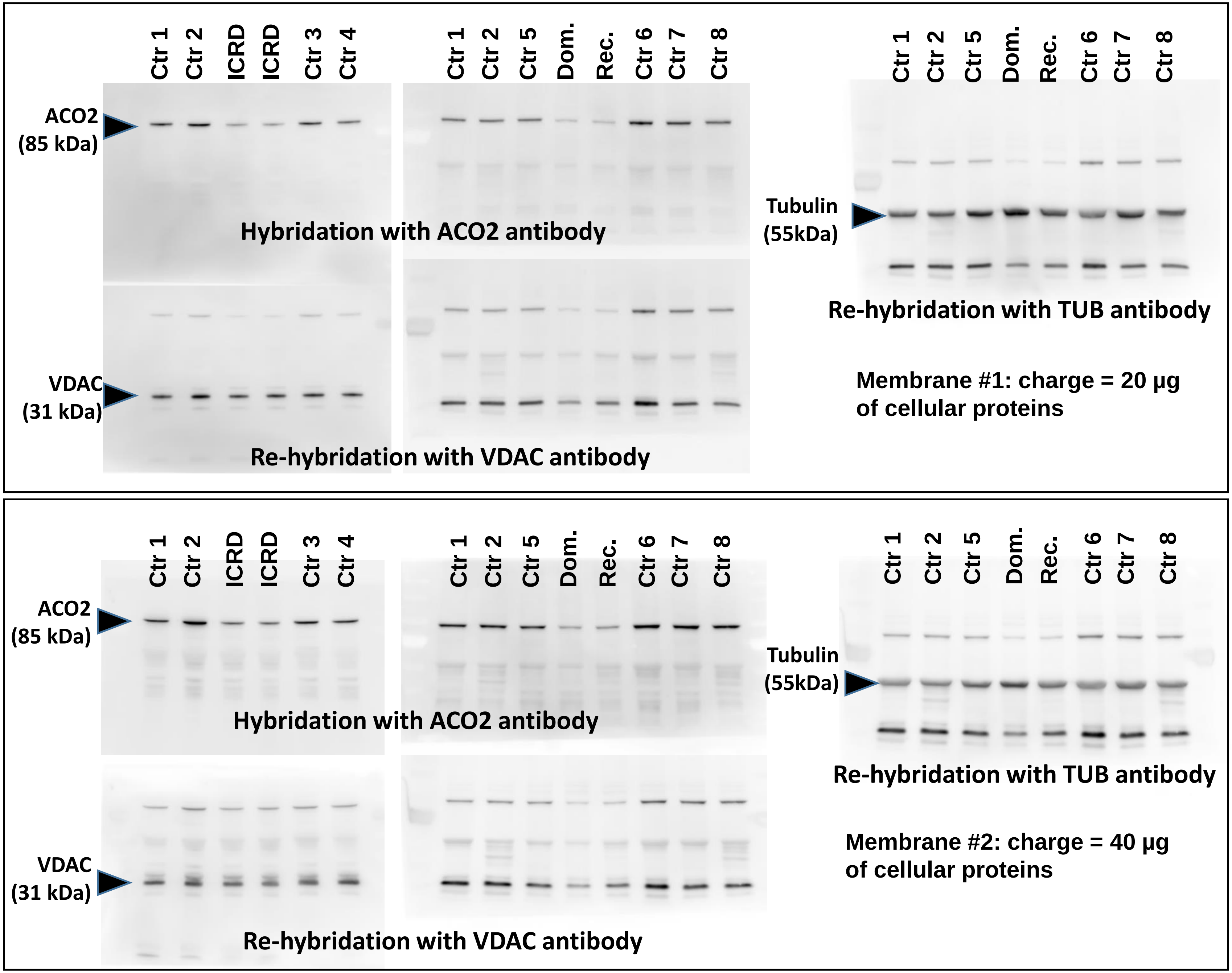

Assessment of ACO2 abundance in fibroblasts by Western Blot

Supplementary Figure 1

Appendix:

The European ION Group is composed of the following collaborators:

- **Francesca Bisulli**: Unit of Neurology, Department of Biomedical and NeuroMotor Sciences (DIBINEM), University of Bologna, Bologna, Italy; IRCCS Institute of Neurological Sciences of Bologna, Bellaria Hospital, Bologna, Italy.

- **Catherine Vignal**: Neuroophthalmology Department, Rothschild Ophthalmologic Foundation, Paris, France

- **Sabine Defoort-Dhellemmes, Isabelle Drumare-Bouvet**: Exploration of Visual Function and Neuro-Ophthalmology Department, Lille University Hospital, Rue Emilie Laine, Lille Cedex, France.

- **Yaumara Perdomo Trujillo, Helene Dollfus**: Centre de référence pour les Affections Rares en Génétique Ophtalmologique, CHU de Strasbourg, Strasbourg, France.

- **Sylvie Odent**: Department of Clinical Genetics, CHU de Rennes, Rennes University, CNRS IGDR (institut de génétique et développement de Rennes), UMR6290, Rennes, France.

- **Carmen Ayuso**: Department of Genetics, Health Research Institute-Jiménez Díaz Foundation, University Hospital (IIS-FJD-UAM), Madrid, Spain.

- **Dan Milea**: Singapore Eye Research Institute, Singapore National Eye Centre and Duke-NUS, Singapore, Singapore.

- **Astrid Plomp**: Department of Clinical Genetics, Amsterdam University Medical Centre, AMC, Amsterdam, The Netherlands

- **Arturo Carta**: Medical and Surgery Department (DIMEC), University of Parma, Parma, Italy

- **Anna Maria De Negri**: Azienda Ospedaliera San Camillo-Forlanini, Rome, Italy

- **Costantino Schiavi**: Ophthalmology Unit, S.Orsola-Malpighi University Hospital, University of Bologna, Bologna, Italy

- **Maria Lucia Cascavilla**: Department of Ophthalmology, University Vita-Salute, IRCCS Ospedale San Raffaele, Milan, Italy

- **Piero Barboni**: Department of Ophthalmology, University Vita-Salute, IRCCS Ospedale San Raffaele, Milan, Italy; Studio Oculistico d'Azeglio, Bologna, Italy,

- **Klaus Rüther** : Augenarztpraxis, Dorotheenstraße, Berlin, Germany

- **Wolf A Lagrèze**: Eye Center, Medical Center, Faculty of Medicine, University of Freiburg, Freiburg, Germany.
